# Immune-Pineal Axis: Nuclear Factor κB (NF-κB) Mediates the Shift in the Melatonin Source from Pinealocytes to Immune Competent Cells

**DOI:** 10.3390/ijms140610979

**Published:** 2013-05-24

**Authors:** Regina P Markus, Erika Cecon, Marco Antonio Pires-Lapa

**Affiliations:** Laboratory of Chronopharmacology, Department of Physiology, Institute of Bioscience, University of São Paulo, São Paulo 05508-900, Brazil; E-Mails: erika.cecon@usp.br (E.C.); marcolapa@usp.br (M.A.P.-L.)

**Keywords:** immune-pineal axis, nuclear factor κB—NF-κB, melatonin, pineal gland, innate immune response, glucocorticoid, macrophage

## Abstract

Pineal gland melatonin is the darkness hormone, while extra-pineal melatonin produced by the gonads, gut, retina, and immune competent cells acts as a paracrine or autocrine mediator. The well-known immunomodulatory effect of melatonin is observed either as an endocrine, a paracrine or an autocrine response. In mammals, nuclear translocation of nuclear factor κ-light-chain-enhancer of activated B cells (NF-κB) blocks noradrenaline-induced melatonin synthesis in pinealocytes, which induces melatonin synthesis in macrophages. In addition, melatonin reduces NF-κB activation in pinealocytes and immune competent cells. Therefore, pathogen- or danger-associated molecular patterns transiently switch the synthesis of melatonin from pinealocytes to immune competent cells, and as the response progresses melatonin inhibition of NF-κB activity leads these cells to a more quiescent state. The opposite effect of NF-κB in pinealocytes and immune competent cells is due to different NF-κB dimers recruited in each phase of the defense response. This coordinated shift of the source of melatonin driven by NF-κB is called the immune-pineal axis. Finally, we discuss how this concept might be relevant to a better understanding of pathological conditions with impaired melatonin rhythms and hope it opens new horizons for the research of side effects of melatonin-based therapies.

## 1. Introduction

The movements of rotation and revolution of the Earth impose cyclic environmental conditions on all living organisms. The ability to anticipate the alternation between night and day and between the seasons is a crucial feature in order to live with this cyclic condition. Mammals have developed a complex endogenous oscillatory system responsible for the anticipation and adaption of physiological functions to environmental changes. The central clock located in the suprachiasmatic nuclei oscillates in a circadian manner. This system relies on the retina, which senses the presence of light to adjust the central clock to environment lighting and on neuroendocrine output to synchronize the endogenous rhythms to the environmental light/dark cycle. The endocrine output of the clock is the nocturnal synthesis of melatonin, which occurs in both diurnal and nocturnal animals. In the 1980s, Russ Reiter defined this hormone as both a clock and a calendar, as its daily rhythm marks the 24 h of the day, and the duration of its peak allows the organism to distinguish between summer and winter (*i.e.*, long and short days) [1].

Melatonin is a pluripotent molecule that acts as the chronobiological hormone and as a cytoprotective mediator. Melatonin as well as its metabolites are important free radical scavengers that reduce deleterious oxidant activities and contribute to the regulation of the redox state of cells [2]. Initially, this mechanism was considered as the basis for the cytoprotective effect of melatonin, but a growing number of studies now indicate a more complex role of the molecule in the defense system of the body. Melatonin is able to modulate immune responses by inhibiting the activation of inflammatory processes and regulating the proliferation and activity of immune competent cells [3–6]. In fact, there are a great number of papers that show the importance of melatonin during innate and acquired immune responses [3–5,7]. Another line of research links the defense response to chronobiological events [8–12]. Here, we focus on the role of nuclear factor κ-light-chain-enhancer of activated B cells (NF-κB) in coordinating the pineal and extra-pineal sources of melatonin that contribute to the development and resolution of an innate immune response. This approach leads to an integral vision of the numerous roles of melatonin, and allows for a discussion of putative collateral effects of exogenous melatonin administered in conditions where the mounting of an inflammatory response is mandatory for the recovery of the patient.

The mounting of an innate immune response requires an immediate recruitment of leukocytes to the site of the lesion. The maintenance of circulating leukocytes without leakage to healthy tissues and the termination of migration during the recovery phase is a multi-mediated process, and melatonin in the pM range contributes to impaired leukocyte migration [13,14]. In turn, when the immune system is activated by infection or danger signals from necrotic tissues [15,16], melatonin production is interrupted in order to allow migration of leukocytes to the site of lesion, which is necessary to mount an inflammatory response. In an attempt to defeat this aggression, neutrophils are recruited to the site of lesion where they release toxic substances, such as nitric oxide, as well as cytokines and chemokines. These first defense cells then recruit professional phagocytes in order to remove pathogens and cell debris.

The first released cytokines act on pinealocytes and transiently inhibit nocturnal melatonin synthesis [17,18]. In addition, activated mononuclear and polymorphonuclear cells are able to synthesize melatonin, thereby contributing to the recovery phase by reducing the oxidative stress and then increasing macrophage phagocytic ability [19,20]. The coordination of this shift between pineal and extra-pineal melatonin suggests the existence of an immune-pineal axis, which is triggered by pathogen-associated molecular patterns (PAMPs), such as lipopolysaccharides (LPS) from gram-negative bacteria, zymosan from fungi, or double stranded RNA from viruses as well as danger-associated molecular patterns (DAMPs), such as amyloid β peptide (Aβ), heat-shock proteins, uric acid, ultra violet light, and tissue debris [21,22].

The signaling cascades triggered by PAMPs and DAMPs lead to nuclear translocation of the transcription factor nuclear factor κ-light-chain-enhancer of activated B cells (NF-κB), which binds to the promoters of target genes of proteins that mediate the innate immune response. The first proteins synthesized are those related to the pro-inflammatory phase, such as pro-inflammatory cytokines, adhesion molecules, and enzymes [inducible nitric oxide (iNOS); cyclooxygenase 2 (COX2)], while the late-response target genes mediate the anti-inflammatory phase, such as the gene that codes for the inhibitory κ B proteins. In the context of the immune-pineal axis, we observed that transcription factor NF-κB is the central player that allows the synchronization between the activation of the immune system and the shift of melatonin sources from the pineal gland to the locally activated immune competent cells. Finally, melatonin inhibits NF-κB activation and plays a direct role in ending the process.

## 2. NF-κB Signaling

NF-κB was named according to its first functional role described and comprises a family of transcription factors that share a REL homology domain. The NF-κB family is evolutionary conserved and includes five subunits [NFκB1 (p50), NFκB2 (p52), RelA (p65), RelB, and cRel] that form homo- or heterodimers. NF-κB dimers are maintained in the cytoplasm through the binding to an inhibitory protein, named inhibitory κ B (IκB). Two distinct pathways lead to the release of the NF-κB dimers from IκB, exposing the nuclear localization signal (NLS) domain and promoting nuclear translocation of NF-κB. Pro-inflammatory cytokines, viruses, and activation of Toll-like receptors (TLRs) trigger the canonical pathway, while the non-canonical pathway is linked to developmental stimuli. Communication between these two pathways provides proper internal fine-tuning of the NF-κB system [23].

The five NF-κB proteins are subdivided in two distinct groups: RelA, RelB, and c-Rel share a transactivation domain (TAD) located in the C-terminus, while the NF-κB1 (p50) and NF-κB2 (p52) are processed from larger precursors (p105 and p100, respectively) that do not possess a TAD [24]. Therefore, homodimers of p50/p50 or p52/p52 inhibit gene transcription, while dimers composed of at least one TAD-positive subunit positively regulate gene expression. NF-κB target genes are mainly early-response genes that mediate cell stress response, innate and adaptive immunity, development, and differentiation [25]. Besides being a central transcription factor for immune-related responses, NF-κB and abnormal activation of its pathway are linked to several diseases, such as cancer, neurodegenerative disorders, and chronic inflammatory diseases [26–28].

Activation of NF-κB through the canonical pathway involves the activation of the IκB kinase complex, which is formed by three distinct proteins (IKKα, IKKβ, and IKKɛ, also called NF-κB essential modifiers—NEMO), and the phosphorylation of IκB, which is then ubiquitinated and processed by the 26S proteasome. Unmasking the NLS domain enables nuclear translocation of NF-κB dimers. The most common dimers activated by the canonical pathway are p50/p50 and p50/RelA; the former leads to inhibition of gene transcription, while p50/RelA dimers contain one TAD and transcribe genes linked to the innate immune response. Cell differentiation or developmental stimuli activate the non-canonical pathway, which promotes nuclear translocation of the p52/RelB dimer. Ligand binding of some membrane receptors activates NF-κB-inducing kinase (NIK), leading to proteasomal processing of p100 into p52, which can then form a dimer with RelB. However, these two pathways are not totally independent, as IKKα also phosphorylates NIK, resulting in its degradation [29–32].

The specific roles of the different NF-κB dimers are still unclear and remain under evaluation. Recently it has been shown that besides transducing infective stimuli, NF-κB activation also plays a role in the survival of sepsis [33]. These two functions are transduced by different NF-κB dimers. The classical p50/RelA is responsible for the initial response, while knocking out c-Rel enhances mortality due to polymicrobial sepsis. In mice, c-Rel controls the transcription of genes involved in host survival and lipid metabolism. NF-κB has a dual function in pathological conditions associated with neurodegeneration: it can either induce neuron death or survival. Post-ischemic brain damage is associated with activated NF-κB. Dimers that contain c-Rel lead to neuronal protection, while those that contain p50/RelA are determinants of cell death [34,35]. Nevertheless, the homodimers RelA/RelA and p50/p50 are also related to neuronal protection, since overexpression of RelA/RelA decreases apoptosis in primary cortical neurons and *nfκB1* null mice exhibit increased cell death under conditions that mimic neurodegenerative diseases [36,37].

Another important topic that is poorly understood is the temporal profile of the NF-κB effect, as it can rapidly reach promoters that are constitutively accessible as well as induce the late recruitment of promoters that require stimulus-dependent acetylation to be accessible to NF-κB [38]. This might be due to the oscillatory pattern of NF-κB nuclear translocation, which can persist even for hours after the first stimulus [39]. In addition, the affinity of NF-κB dimers to DNA varies according to the subunits that compose the dimers. Accordingly, the gene that codes the anti-inflammatory protein IL-12 has a high affinity for c-Rel, and it is only translated when dimers containing this subunit are activated [40]. It is interesting to note that c-Rel is related to the recovery phase of the process, inducing the synthesis of anti-inflammatory cytokines [38] and protecting mice against sepsis [33]. In summary, NF-κB is a pivotal transcription factor that regulates the time course of the innate immune response, thereby controlling the on/off timing of genes related to the pro-inflammatory and recovery phases.

## 3. Melatonin and the Regulation of NF-κB Activation

The inhibition of the NF-κB pathway by melatonin was first reported in 1995 in HELA cells stimulated with the pro-inflammatory cytokine tumor necrosis factor (TNF) and ionizing radiation [41]. Several studies with cells, tissues, and whole animals have shown that melatonin inhibits NF-κB either by oxidative stress or PAMPs. Melatonin reduces NF-κB activation in macrophages [42], T cells [43,44], RAW 264.7 cell line [45–47], neuronal tissue and cell culture [47–49], liver [50], kidney [51,52], lung [53], and heart [54]. Studies based on models of inflammatory disease, such as inflammatory bowel disease [55], colitis [56], chronic gastric ulceration model in mice [57], and experimental diabetic neuropathy [58] as well as inflammatory processes, such as hyperalgesia associated with inflammation [59], spinal cord trauma [60], and fulminant hepatic failure [50] in rats describe a reduction of inflammatory output by melatonin. In addition, when evaluated, the anti-inflammatory effect of melatonin was shown to be mediated by the inhibition of NF-κB nuclear translocation [42,49,54,61–63].

More recently, some data indicate a more complex scenario, as melatonin was shown to activate NF-κB in U937 cells, which are a lineage derived from monocytic human cells [64,65]. This dual effect of melatonin on NF-κB activation is quite interesting, since it is linked to the ability of melatonin to induce angiogenesis during wound healing [66,67]. An evaluation of the effect of melatonin during the process of wound healing showed that it reduced iNOS activity during the pro-inflammatory phase, while it increased its activity during the granulation tissue formation, favoring angiogenesis and healing [67]. This apparent controversial result could be reconciled through a better understanding of the role of the different subunits of NF-κB in each phase of the defense response. However, currently we can only hypothesize that melatonin is able to both inhibit and activate NF-κB nuclear translocation, and that the inhibition is linked to the blockage of the pro-inflammatory phase of the inflammatory response.

In summary, most of the studies in the literature indicate that the anti-inflammatory effect of melatonin is mediated by inhibition of NF-κB activation. However, it is important to note that most of these studies are related to the pro-inflammatory phase of the response or to inflammatory diseases, and thus the role of melatonin in the recovery phase has not as yet been sufficiently explored.

## 4. NF-κB and the Pineal Gland

The pineal gland is the endocrine arm of the chronobiological system in mammals. The synthesis of the dark hormone melatonin is directly controlled by the central biological clock located in the suprachiasmatic nuclei. Darkness is translated to the pineal gland by sympathetic inputs. Melatonin biosynthesis is stimulated by noradrenaline via a combination of β- and α1-adrenergic receptors. This results in rises of cAMP, cytosolic calcium and activation of PKA, PKC and CaMK. The key enzyme, arylalkylamine *N*-acetyltransferase (AA-NAT) can be regulated at the transcriptional or at a posttranslational level [68]. Upregulation of AA-NAT gene expression is mediated by pCREB that binds to CRE in the AA-NAT promoter. This mechanism prevails in nocturnally active rodents, although the additional posttranslational control also exists. In primates and ungulates, AA-NAT is mainly posttranslationally regulated. Phosphorylation of the enzyme by PKA or PKC allows the association with 14-3-3 proteins (isoforms ζ or ɛ). The moderately stable pAA-NAT/14-3-3 complex prolongs the lifetime of the AA-NAT protein sufficiently to allow a substantial nocturnal increase in active enzyme.

The pineal gland can be considered a unique organ in many aspects, as it is a gland derived from the roof of the embryonic forebrain, and in the adult brain it constitutes the main part of the epithalamus together with the habenular nuclei [69]. The pinealocytes, which constitute 90% of the cells that make up to the pineal gland, are modified neurons that originate from neuronal-germ cells, and astroglia as well as microglia are also present in the pineal gland. Finally, the pineal gland is a circumventricular organ that provides a gateway into the central nervous system.

In 2005, we reported on a study designed to evaluate the effect of corticosterone on melatonin synthesis in the rat pineal gland and found that glucocorticoids reduce NF-κB content in nuclear extracts of rat pineal glands maintained in culture for 48 h [70]. In addition, corticosterone increases the synthesis of melatonin induced by stimulation of β-adrenoceptors, while TNF impairs it [17]. These data strongly suggest that the pineal gland is an integral part of the innate immune response, but also suggest a putative role of NF-κB in the daily physiology of the pineal gland, since we observed constitutive NF-κB activation in non-stimulated glands [71].

In the brain, NF-κB is not only related to innate and acquired immune responses, but is also essential for neuron development and survival [36]. NF-κB plays a role in neurite outgrowth [72], cell fate determination [73], functional circuit formation, and tissue homeostasis [74]. In the rat pineal gland, NF-κB is constitutively translocated to the nucleus during daytime and a sharp decrease in its nuclear content occurs immediately after the lights are turned off [71]. This decrease is maintained in free-running conditions, indicating that it is driven by endogenous rhythmicity and corresponds to the entrance of subjective night. In animals exposed to light-dark cycles of 12 h:12 h, low levels of nuclear NF-κB are maintained during the entire dark phase, and then begin to increase during the daytime, attaining a maximal concentration just before lights are turned off. In animals maintained in constant darkness, the significant drop of NF-κB upon entry of subjective night is still observed, however, the nuclear level of this transcription factor begins to increase much earlier than the entrance of the subjective day. Interestingly, blockage of β-adrenoceptors does not impair the dark-induced decrease in nuclear NF-κB, but rather allows an earlier increase, attaining daytime values during the scotophase. Because melatonin can block NF-κB activation [75,76], we suggest that the maintenance of a low concentration during nighttime is due to melatonin autocrine effects on pinealocytes, which indeed was confirmed in *in vitro* experiments. As for the free-running animals, the amount of melatonin synthesized is much lower than that observed in animals maintained under a light-dark cycle and most likely not sufficient to block NF-κB activation [71].

Another important point is that in the absence of PAMPs or DAMPs challenge (in healthy conditions), only the p50/p50 dimer is present in the nuclei of pinealocytes. This dimer has no TAD, and therefore its binding to DNA should inhibit gene transcription. This hypothesis is supported by data showing a high level of NF-κB in the nuclei of a daytime gland that sharply decreases at the entrance of nighttime, which would allow for the induction of gene transcription by other transcription factors. In the case of AA-NAT, noradrenaline-induced cyclic AMP-regulated transcription factor (CREB) phosphorylation would induce the transcription of the gene. Thus, the repressive p50/p50 NF-κB dimer could be important in order to repress the transcription of AA-NAT during the daytime in rats.

In chronic inflammatory conditions, the nocturnal melatonin synthesis depends on a balance between pro-inflammatory mediators and cortico-adrenal hormone action on the pineal gland. Accordingly, adrenalectomy blocks the nocturnal melatonin peak in animals chronically inflamed with BCG [77], while intrapineal perfusion of corticosterone increases nocturnal melatonin synthesis [17]. Since NF-κB signaling is the pivotal pathway in the innate immune response, NF-κB activation should play a critical role in this balance and the pineal gland should possess all of the molecular tools needed to play an integral role in innate immune responses. Indeed, pinealocytes, microglia, and astrocytes of the rat pineal gland express TLR4 and TNF membrane receptors, which signal through the NF-κB pathway [18,78]. The membrane protein CD14, which is necessary for the binding of LPS to TLR4, is also present. LPS induces the activation of the NF-κB pathway through the stimulation of TLR, which leads to the nuclear translocation of NFKB1 (p50) and RelA, but not of NFKB2 (p52), RelB, or c-Rel. The two NF-κB dimers detected in the nuclear extract of pineal glands are p50/p50 and p50/RelA, indicating that activation of the NF-κB pathway may result in both the induction and repression of a separate package of genes simultaneously. The synthesis of TNF following LPS-induced NF-κB activation is restricted to pineal microglia, while in pinealocytes the stimulation of LPS and TNF increase the expression of TNFR1 receptors. Therefore, the activation of the NF-κB pathway in the pineal gland is mediated by a direct interaction of LPS with CD14/TLR4 receptors as well as by the late interaction of microglia-derived TNF with TNFR1.

We have identified several clinical conditions that have phenotypic features of these molecular events described in the pineal gland following an innate immune response. Patients with mastitis [19] or those undergoing a surgical procedure, such as caesarean section [79] or hysterectomy [80], exhibit a reduction or suppression in their normal nocturnal melatonin increase. The concentration of melatonin in the colostrum (third day milk) of healthy women presents a daily rhythm. However, when the mother has mastitis, which is an acute inflammation induced by suckling, no difference between daytime and nighttime melatonin was detected [19]. The same profile was observed in women that delivered by caesarean section [79]. In both cases (mastitis and caesarean section), the level of nocturnal melatonin had an inverse correlation with the level of TNF, strongly suggesting that this pro-inflammatory cytokine impairs the natural increase in nocturnal melatonin levels. Fifteen days after caesarian section, which is the point when no TNF can be detected, the normal daily rhythm of melatonin was restored. [79]. In that study it was shown that one mother had a slower reduction in TNF levels and only restored the daily rhythm of melatonin 20 days after the delivery. Impairment in the melatonin rhythm has also been observed in other pathological conditions, such as in ischemic stroke [81,82], psychiatric diseases [83–85], and neurodegenerative disorders [86,87]. Further evaluation of these observations with knowledge of the immune-pineal axis concept may provide a better understanding of the pathophysiological processes involved in each case as well as optimization of therapeutic melatonin administration protocols (reviewed by [88,89]). Taken together, these data indicate that pro-inflammatory and anti-inflammatory mediators regulate pineal gland activity through the NF-κB pathway. Moreover, this mechanism could explain the transient dysfunction in the temporal organization that accompanies the sickness behavior observed in innate immune or acute inflammatory responses.

## 5. NF-κB and the Regulation of Melatonin Synthesis in Extra-Pineal Tissues

Many organs and cells, besides the pineal gland, have the ability to synthesize melatonin. Melatonin production has been demonstrated in the retina, gastrointestinal tract, skin, immune, and hematopoietic cells, under specific conditions. The synthesis of melatonin by the retina follows a daily profile and is involved in chronobiological responses as well as local cellular protection [90]. During development, other brain areas beyond the pineal gland produce melatonin [91]. The synthesis of melatonin by enterochromafin cells of the gastrointestinal tract also occurs under physiological conditions, but it is mainly related to the periodicity of food intake, rather than environmental influence (for review, [92,93]). In fact, the amount of melatonin in the gastrointestinal tract, including liver and pancreas, reaches concentrations that are 400 times higher than that in the blood. The lack of a direct correlation between blood and gastrointestinal melatonin is due to the efficient conversion to hydrosoluble compounds (reviewed by [2]). Gastrointestinal melatonin is a local protector against injuries caused by an excess of acid and also contributes to the healing of ulcerative processes [93].

Although initially doubted, there is now a large amount of data confirming that cells from the immune system synthesize melatonin, which plays a paracrine, autocrine, and intracrine role in the regulation of innate and acquired immune responses [21,94,95]. Both circulating immunological cells and progenitor cells in the bone marrow express the enzymes involved in melatonin synthesis [AA-NAT and acetylserotonin methyltransferase (ASMT)]. Melatonin production has been detected in human lymphocytes [95], rat resident peritoneal macrophages [96], human mononuclear and polymorphonuclear cells from the colostrum [19], mast cells [43], murine lineage macrophages RAW 264.7 [20], glial cells [97], and many others [6]. All these cell types also express melatonin receptors, which mediate some of the paracrine and autocrine effects of the molecule [20,43,44].

In mammals, two melatonin high affinity G-protein coupled receptors (GPCRs) have been identified, named MT1 and MT2. Both are able to interact with Gi or Gq proteins upon activation, thereby triggering intracellular cascades that involve inhibition of cyclic AMP production or release of intracellular calcium from intracellular compartments, respectively. Other melatonin binding sites include intracellular molecules, such as the calcium-binding protein calmodulin, the enzyme quinone reductase 2, cytoskeletal and scaffold proteins, and the nuclear (orphan) receptors of the family ROR and RZR, which are related to retinoic family receptors. The review of the molecular interaction of the receptors and the signaling cascades for melatonin receptors is outside the scope of the present paper, and can be found elsewhere (reviewed by [98,99]).

One of the most investigated mechanisms of action of melatonin in the innate immune response involves its antioxidant properties. Melatonin and its metabolites are capable of scavenging free radicals and inducing the expression of antioxidant enzymes, resulting in a protective effect against oxidative damage in tissues and contributing to the maintenance of a redox balance within the cell under adverse conditions [100]. In neutrophils, melatonin and its oxidation product N1-acetyl-N2-formyl-5-methoxykynuramine (AFMK) inhibit the LPS-mediated production of TNF and interleukin-8 (IL-8), which are cytokines important for leukocyte recruitment, thus representing an anti-inflammatory role of melatonin [101]. Melatonin increases the phagocytic activity of human colostral mononuclear cells, and this effect is dependent on MT2 receptor activation [102], while expression of the interleukin 2 (IL-2) and IL-2 receptor genes are mediated by MT1 membrane melatonin receptors [103]. Nevertheless, the activation-associated death of lymphocytes, which is an apoptotic process [100], is independent of melatonin G-coupled receptors [44,104]. The effects of melatonin mediated by membrane receptors are induced at lower concentrations and are in agreement with those found in the nocturnal plasma of healthy subjects. Therefore, identifying the mechanisms of action of melatonin on immune competent cells provides evidence for effects linked to healthy or unhealthy conditions. An interesting example of this was observed in a longitudinal study of women that delivered by caesarean section, whose acute increase in TNF led to impairment in both melatonin and IL-2 rhythms, while both rhythms were simultaneously restored approximately 20 days after the surgery [79]. Because melatonin-dependent regulation of IL-2 production by lymphocytes is mediated by membrane receptors [105], it is not surprising that this regulatory mechanism is sensitive to circulating melatonin. In addition, it is important to note that Maestroni and colleagues [105] showed a modulation of cytokines, and in particular IL-2, by melatonin, which was recently reviewed in a discussion of the implications of melatonin in the immunomodulation of seasonal diseases [106].

Melatonin production by activated macrophages is dependent on NF-κB nuclear translocation [20]. Although NF-κB inhibits *Aa-nat* transcription in pinealocytes, it leads to the opposite effect on macrophages. The fact that the pineal gland and the immune competent cells share the same transduction pathway upon activation of an immune response (NF-κB), but result in opposite effects on the same target gene (*Aa-nat*), led to the conclusion that NF-κB is the key component for the immune-pineal axis [21,22]. Thus, this pivotal transcription factor is responsible for the shift between pineal and extra-pineal melatonin sources during inflammatory responses.

## 6. The Immune-Pineal Axis: The NF-κB Pathway Coordinates Pineal and Extra-Pineal Melatonin Synthesis

Macrophages challenged with *Escherichia coli*, LPS, or zymosan synthesize melatonin due to activation of the NF-κB pathway, since blocking the proteasome degradation of IκB or the binding of NF-κB dimers to DNA impairs melatonin synthesis [20,102]. According to an *in silico* study, the promoter and the first intron of the gene that codes for AA-NAT contain κB sequences, which suggests a putative regulatory role of NF-κB on AA-NAT expression [21]. More recently, we generated RAW 264.7 macrophage cell lines from mice expressing a red fluorescent reporter protein under the control of κB elements present in the promoter and in the first intron of the *Aa-nat* gene. Treatment of these cells with LPS induced *Aa-nat-REPORT* transcription and melatonin synthesis, and both effects were blocked by pharmacological and siRNA-mediated inhibition of RelA and c-Rel NF-κB activity [20]. It is interesting to note that in the pineal gland, the dimer p50/p50 is responsible for inhibiting *Aa-nat* transcription, while the p50/RelA dimer mediates the induction of TNF by pineal microglia cells. Otherwise, activation of c-Rel containing dimers is linked to the positively regulation of *Aa-nat* transcription. Thus, depending on the activation of specific NF-κB dimers, melatonin synthesis can be turned on or off.

As mentioned before, melatonin synthesized by RAW 264.7 cells has an important autocrine role that potentiates phagocytosis by stimulating melatonin G-protein coupled receptors. This response, obtained at the nM range, was further confirmed in human colostral mononuclear cells challenged with zymosan [102]. The zymosan-induced TLR2-NF-κB pathway activation results in melatonin production that acts through MT2-melatonin receptors and increases the expression of the membrane protein dectin-1, which is crucial for fungal phagocytosis. Moreover, melatonin in the μM-mM range, which is not toxic for RAW 264.7 cells [107], inhibits TNF, IL-1β, IL-6, IL-8, and IL-10 synthesis and attenuates the up-regulation of COX-2 and iNOS [108]. These effects are due to melatonin-induced inhibition of the expression of myeloid differentiation factor 88 (MyD88), which is one of the first steps in the canonical cascade that links TLR4 to NF-κB activation. Thus, melatonin produced by macrophages plays a double role: First, in the pM-nM range, it enhances their phagocytic capacity, and then at higher concentrations, it contributes to returning the cells to the quiescent state by impairing NF-κB activity itself, thereby avoiding excessive spreading of inflammatory mediators.

## 7. Concluding Remarks

Inflammation is triggered when innate immune cells detect infection or tissue injury. Surveillance involves the recognition of PAMPs and DAMPs, which induce the nuclear translocation of NF-κB and other transcription factors that control the expression of specific genes related to recruitment and activation of leukocytes, critical cells for eliminating foreign particles and host debris. In healthy conditions the migration of leukocytes from the circulation to tissues is not desirable, as it can result in an unnecessary inflammatory response and subsequent tissue damage [109]. Therefore, surveillance also involves the availability of a great number of leukocytes in the circulation in order to notify the immune system of the presence of DAMPs and PAMPs, which then activate the innate immune response and induce migration to the site of the lesion. Under physiological conditions, plasma melatonin reduces the expression of adhesion molecules in endothelial cells, resulting in an impairment of leukocyte migration from the blood to healthy tissues [13,110,111], thus playing a role in surveillance. It is interesting to note that cultures of endothelial cells obtained from animals euthanized at nighttime also express lower levels of adhesion molecules and iNOS [111]. This effect is mediated by inhibition of NF-κB, and therefore genes involved in mounting an innate immune response have lower expression levels at nighttime. The bidirectional communication between the pineal gland and immune competent cells is important to fine-tune the ability of leukocytes to migrate from the circulation during normal or inflamed states [21,22,112,113]. Proper leukocyte migration is achieved in the absence of circulating melatonin due to inhibitory action of NF-κB on pineal hormonal production. The same stimulus and the same transcription factor simultaneously induce local melatonin production by immune competent cells, which plays essential roles in improving immune cell activity, protecting the tissue from further damage by reactive oxygen and nitrogen species, and finally contributing to the termination of the immune response through NF-κB inhibition [7,114]. The blockage of NF-κB activity is essential for restoring pineal synthesis of melatonin. In the anti-inflammatory phase of the innate immune response, the increase in glucocorticoid production also blocks NF-κB activation in the pineal gland, leading to an increase in *AA-NAT* transcription and melatonin synthesis [70,115].

**Figure 1:**
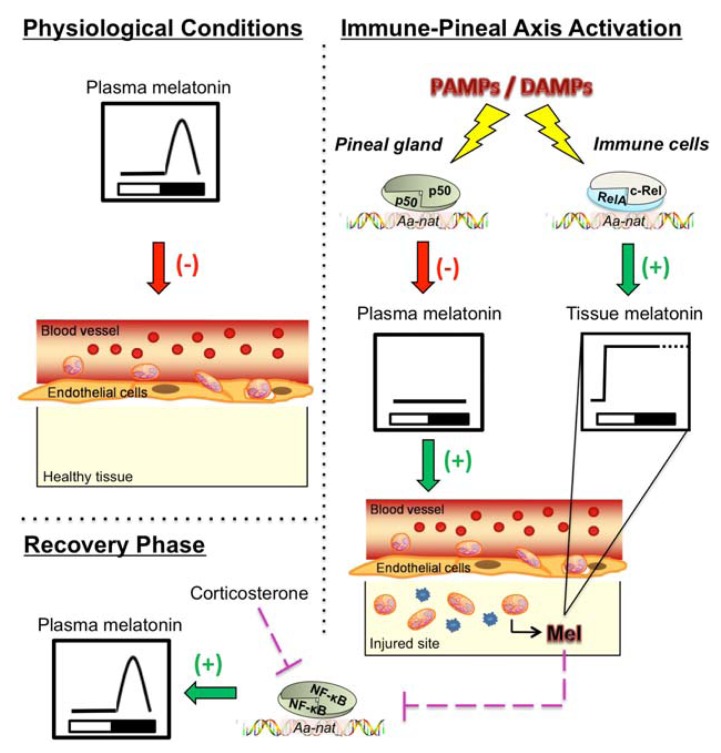
Schematic representation of NF-κB-mediated shift in melatonin sources upon activation of the immune-pineal axis. **Left side**—Physiological conditions: Melatonin synthesized at nighttime by the pineal gland impairs leukocyte migration. **Right side**—Interaction of PAMPs or DAMPs with their receptors triggers the nuclear translocation of NF-κB both in the pinealocytes and macrophages. NF-κB dimers bind to κB elements located in the gene that codes for AA-NAT and control its transcription in a tissue-specific manner. In the pinealocytes, the homodimer p50/p50 blocks *AA-NAT* transcription, while in macrophages the heterodimer RelA/c-Rel induces *AA-NAT* transcription and local melatonin production. **Bottom**—Melatonin also participates in the resolution phase, as it reduces the nuclear concentration of NF-κB, thereby reducing the transcription of genes involved in the pro-inflammatory phase of the innate immune response. During this phase, the effect of melatonin and corticosterone are synergic, as both reduce the nuclear content of NF-κB. In the pineal gland, corticosterone increases the noradrenaline-induced melatonin production *in vitro*, and nocturnal melatonin levels subsequently rise in rats [70,115] (see text for further details).

In summary, the transcription factor NF-κB is the main factor responsible for the shift between pineal and extra-pineal production of melatonin. Based on the cellular microenvironment, NF-κB inhibits (pinealocytes) or induces (macrophages) the transcription of the key enzyme in melatonin synthesis—AA-NAT. This coordinated alteration in the source and in the function of melatonin during an innate immune response has been termed the “Immune-Pineal Axis” [21], and is schematically represented in Figure 1. The importance of melatonin and the pineal gland for the immune system is well documented regarding both the daily and seasonal variation in immune functions as well as the immunomodulatory effects of melatonin during an innate immune response. The former is related to the chronobiotic effects of melatonin, while the latter effect is mainly due to anti-inflammatory and anti-oxidant properties of melatonin and its metabolites [7,100]. However, the integration of pineal and extra-pineal melatonin as well as the definition of its role in each phase of an innate immune response is just now being understood. Melatonin should not be administrated when the mounting of a defense response is mandatory, as for example just after a surgery. On the other hand, if melatonin is not produced at the site of lesion, the prognosis of the case is expected to be worse and melatonin administration could be recommended. The advent of the immune-pineal axis concept will certainly contribute to a better understanding of the relationship between the pineal and immune functions. This will also have further implications on the knowledge concerning pathological conditions in which the melatonin rhythm is disrupted as well as on improving therapeutic protocols that involve melatonin administration.
